# Reprogramming the wound microenvironment: identity remodeling strategies for fibroblasts, keratinocytes, and macrophages

**DOI:** 10.3389/fimmu.2026.1892931

**Published:** 2026-07-20

**Authors:** Jing Wang, Min Chen, Jiao Wei, Xiaojun Chen, Chenglong Wang, Huirong Feng

**Affiliations:** 1 Department of Orthopedics, The Affiliated Traditional Chinese Medicine Hospital, Southwest Medical University, Luzhou, Sichuan, China; 2 Department of Hand surgery, The Affiliated Traditional Chinese Medicine Hospital, Southwest Medical University, Luzhou, Sichuan, China; 3 Department of Pediatric Orthopedics, The Affiliated Traditional Chinese Medicine Hospital, Southwest Medical University, Luzhou, Sichuan, China; 4 Luzhou Key Laboratory for Prevention and Treatment of Orthopedic Diseases Through Integrated Traditional Chinese and Western Medicine, Luzhou, Sichuan, China; 5 Mianyang Orthopaedic Hospital, Mianyang, Sichuan, China

**Keywords:** cell reprogramming, epigenetic regulation, fibroblasts, keratinocytes, macrophages, metabolic reprogramming, trained immunity, wound repair

## Abstract

The core pathology of chronic non-healing wounds is the dysfunction of wound-repair cells—a molecular defect that conventional passive therapies can hardly correct at its root. Cell reprogramming techniques, by actively rewriting cell identity, have therefore brought a paradigm shift to wound repair. Herein, we propose a systematic “four-dimensional technology toolbox” for wound cell reprogramming, comprising transcription factor-mediated, small-molecule-induced, epigenetic and metabolic regulation, and nanomaterial-assisted delivery. Using this toolbox as the main thread, we comprehensively integrate three core cell-identity reprogramming strategies: fibroblasts (from a profibrotic scar-forming phenotype to a pro-regenerative repair-competent phenotype), keratinocytes (restoring the endogenous regenerative capacity of keratinocytes to reconstruct the epidermal barrier), and macrophages (from a pro-inflammatory pathological state to a reparative homeostatic phenotype). Remedying the principal weaknesses of existing reviews—overemphasis on technique listing, weak mechanistic integration, and lack of translational critique—we dissect the key molecular mechanisms layer by layer and critically evaluate the core clinical-translation bottlenecks, including safety, spatiotemporal precision, and model systems. Finally, we spotlight emerging frontiers such as single-cell multi-omics navigation, AI-driven temporally programmed smart materials, and trained immunity, and discuss how they propel the field from proof-of-concept toward a precision systems-engineering paradigm of “personalized diagnosis → intelligent sequential delivery → closed-loop healing monitoring”. This work not only offers a novel intervention paradigm for the core challenges of treating chronic non-healing wounds like diabetic foot ulcers, but also delivers a panoramic theoretical framework and practical guidance for precision reprogramming therapy—from fundamental mechanisms to clinical translation.

## 1 Introduction

Chronic non-healing wounds have become a major global public health burden, among them, diabetic foot ulcers account for 15%–25% of chronic wounds and carry a five-year mortality risk as high as 30% (1). The core of impaired healing lies in the dysfunction of repair cells—metabolic disturbances such as hyperglycemia, oxidative stress, and persistent low-grade inflammation lead to diminished fibroblast migration and contraction, impaired keratinocyte re-epithelialization, and imbalanced macrophage phenotypic polarization, ultimately entrapping the wound in a vicious cycle of “inflammatory persistence–repair arrest” (2, 3). Current standard therapies (debridement, anti-infection, revascularization, negative-pressure wound therapy, and moist dressings) are essentially passive supportive measures that cannot actively correct the already dysfunctional resident cell populations; likewise, regenerative strategies including stem cell transplantation, growth factors, and bioengineered skin substitutes have yet to overcome bottlenecks such as low transplanted cell survival, poor functional integration, and difficulties in scalable manufacturing (4–6).

Cell reprogramming technology has fundamentally shifted the intervention paradigm for wound repair. By actively rewriting the “molecular identity” of wound-resident cells, it restores their intrinsic functions or converts them into the cell types required for healing (7). In 2006, Yamanaka et al. demonstrated that fibroblasts could be reprogrammed into induced pluripotent stem cells (iPSCs) using *Oct4, Sox2, Klf4*, and *c-Myc* (*OSKM* factors), proving that cell identity is malleable. However, the iPSC route faces significant clinical hurdles: inherent tumorigenic risks, the complexity of ex vivo expansion and directed differentiation, and immune rejection associated with allogeneic transplantation (8, 9). To circumvent these limitations, two alternative strategies have emerged. The first is direct lineage reprogramming. This approach uses a limited set of lineage-specific transcription factors to convert one cell type directly into another across germ layers, thereby generating repair-competent cells right at the wound site. In a recent proof-of-concept study, *in situ* delivery of *BMI1* and *FGFR2b* via adeno-associated virus serotype 9 (*AAV9*) vectors in diabetic mouse wounds successfully reprogrammed fibroblasts into induced keratinocyte-like cells (iKCs); these iKCs reconstructed a fully stratified epidermal structure and restored barrier function. The second strategy is partial reprogramming. By transiently and periodically expressing *OSKM* factors—or applying specific chemical stimuli—partial reprogramming erases age-or injury-related epigenetic marks while preserving the original cell identity, thereby restoring the tissue’s endogenous regenerative potential (10, 11).

In recent years, most reviews on cell reprogramming and wound repair have focused on a single reprogramming technology (e.g., epigenetic mechanisms of partial reprogramming) or a single cell type (e.g., fibroblast lineage conversion and scar regulation), lacking a systematic integration of multiple technology platforms—transcription factors, small molecules, epigenetic/metabolic regulation, and nanodelivery systems—with multiple cellular targets, including fibroblasts, keratinocytes, and macrophages (112, 113). Moreover, existing epigenetic reviews largely catalog basic mechanisms such as DNA methylation and histone modifications, without critically examining key translational challenges such as delivery vehicle bottlenecks, the cellular heterogeneity that drives reprogramming efficiency, and the requirement for spatiotemporal precision (12). Finally, a disconnect persists between technological advances and cell biology: while single-cell and spatial multi-omics have revealed pronounced heterogeneity in fibroblast subpopulations and macrophage polarization states within chronic wounds, few reviews frame cellular heterogeneity as the central obstacle to reprogramming precision, nor do they explain at the cell-atlas level why the same reprogramming strategy produces markedly divergent effects in different *in vivo* microenvironments (13, 14).

**Figure 1:**
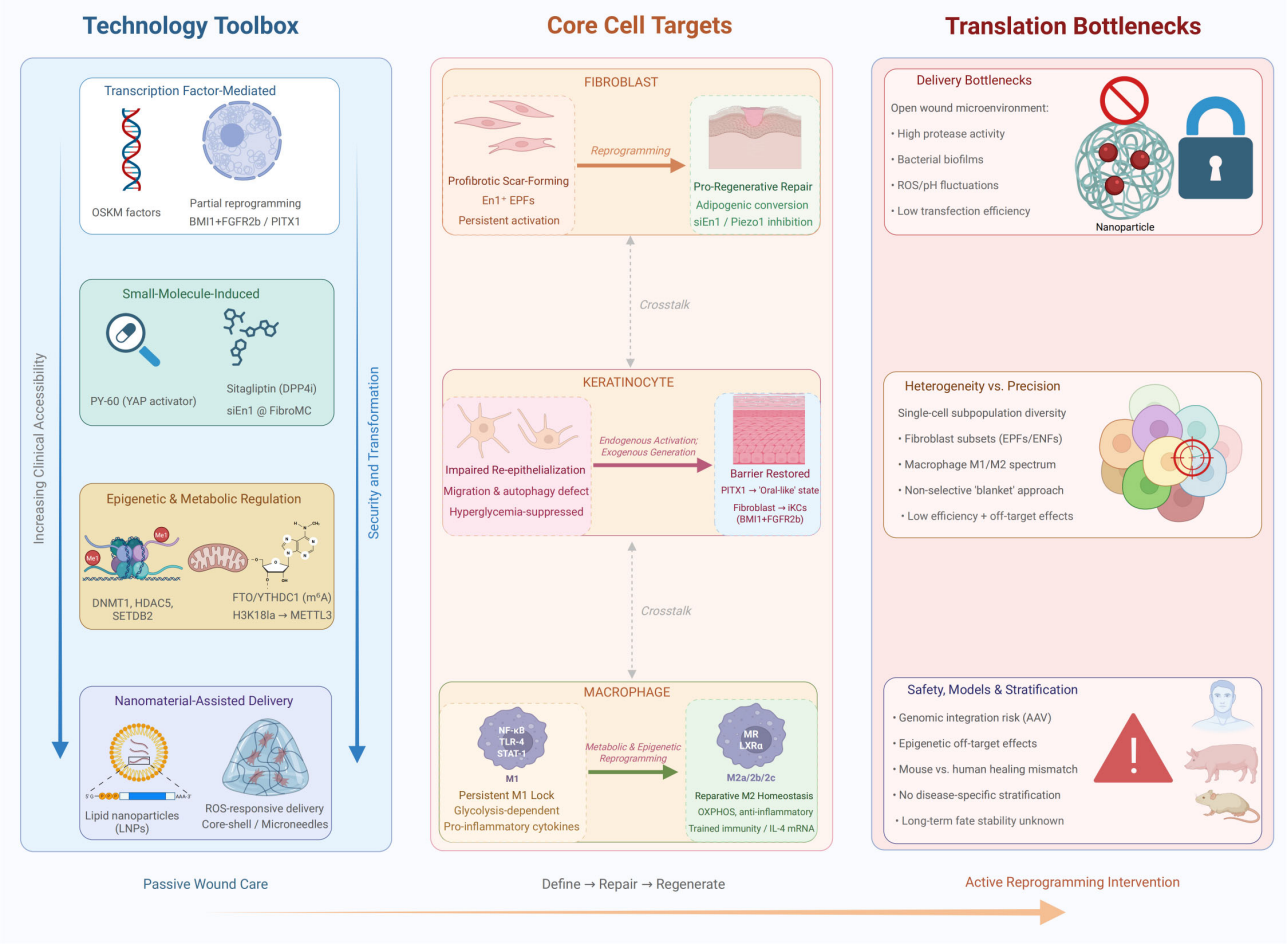
The ‘Technology–Target–Translation’ Triadic Framework for Wound Cell Reprogramming. Schematic overview of the four-dimensional technology toolbox (left) enabling identity reprogramming of fibroblasts, keratinocytes, and macrophages (center) to restore wound repair competence. Clinical translation is currently constrained by three core bottlenecks: delivery limitations, cellular heterogeneity versus precision requirements, and gaps in safety, disease models, and patient stratification (right). (Create with BioRender).

Herein, we provide an integrated, critical perspective built on a triadic framework of “technology platform→cell target→translational bottleneck” (**Figure 1**). This framework remedies the deficiencies of existing reviews, which overemphasize technique enumeration while neglecting mechanistic integration and translational critique. Specifically, we first systematically survey the four-dimensional technology toolbox for wound cell reprogramming. This toolbox encompasses transcription factor-mediated, small-molecule-induced, epigenetic and metabolic regulation, and nanomaterial-assisted delivery. We then focus successively on three pivotal repair cell types—fibroblasts, keratinocytes, and macrophages—dissecting, layer by layer, the molecular mechanisms underlying their dysfunction and the cutting-edge strategies for their identity reprogramming. Finally, we critically assess the field’s central challenges and chart key pathways toward clinical translation. By establishing a conceptual framework that spans from “passive wound care” to “active reprogramming intervention,” this review offers a panoramic perspective. We hope it will serve as a valuable resource for colleagues engaged in both basic research and clinical translation of wound repair.

## 2 Iteration of the reprogramming toolkit

Cell reprogramming technology has advanced from Yamanaka et al.’s 2006 achievement of somatic cell reprogramming using *OSKM* factors to the current capability of bypassing the pluripotent stem cell stage and directly converting cell identity across germ layers *in vivo*—a paradigm evolution from global epigenetic resetting to cell type-specific precision reprogramming. This progress has coalesced into a multidimensional toolbox encompassing transcription factor-mediated, small-molecule-induced, epigenetic and metabolic modulation, and nanodelivery system-assisted approaches (15, 16).

Transcription factor-mediated reprogramming is the technological cornerstone of cell fate manipulation (114). As pioneer transcription factors, *OSKM* occupy regulatory elements within silenced genomic regions early in reprogramming, triggering somatic identity suppression, mesenchymal-to-epithelial transition, and a metabolic shift toward glycolysis (115). This “full reprogramming” route has established the core theoretical framework for cell fate control; however, its clinical translation is hindered by complex ex vivo expansion procedures, the tumorigenic risk of residual pluripotent stem cells, and immune rejection following allogeneic transplantation (9, 17). To circumvent these limitations, partial reprogramming has emerged, which is defined as a strategy that uses transient, cyclic expression of *OSKM* factors to erase age- and injury-related epigenetic marks without fully erasing the original somatic identity, thereby rejuvenating cells to a state of enhanced regenerative capacity. In an *in vitro* study of human epidermal stem cells, transient *OSKM* induction significantly restored self-renewal and proliferative ability while counteracting the accelerated epigenetic clock, with DNA methyltransferase 1 (*DNMT1*) playing a key role in this process (11, 18). Building on this, direct lineage reprogramming (also termed transdifferentiation) offers a more direct approach, defined as the conversion of one terminally differentiated somatic cell directly into another functional cell type using a limited set of lineage-specific transcription factors, without passing through a pluripotent intermediate (19). A landmark *in vivo* study in diabetic mouse wounds exemplifies this approach: *in situ* delivery of *BMI1* and *FGFR2b* via *AAV9* vectors successfully reprogrammed fibroblasts into induced keratinocyte-like cells, reconstructing a fully stratified epidermis (10). Additionally, an *in vivo* mouse skin wound study found that the transcription factor *PITX1* can endow epidermal keratinocytes with a rapidly migratory and proliferative state resembling oral mucosal cells, reshaping the wound’s inflammatory microenvironment and accelerating re-epithelialization—representing another reprogramming paradigm that reactivates endogenous regenerative potential (20).

Small molecules and repurposed drugs represent the most translationally attractive alternative, given their freedom from genomic integration risk, dose controllability, and low cost. Chemical reprogramming employs small molecules to target endogenous signaling pathways and chromatin modifiers, thereby indirectly reconfiguring the gene expression landscape (21). In a porcine wound *in vivo* study and ex vivo human skin study, the *YAP* activator *PY-60* was shown to activate a pro-regenerative transcriptional program, suppress fibroblast transition to a profibrotic phenotype, and promote a near-regenerative repair pattern (22). In the realm of drug repurposing, cell-based and mouse wound *in vivo* studies have demonstrated that the *DPP4* inhibitor sitagliptin can direct fibroblasts to convert into adipocytes, suppressing scar formation at its source (23). One of the most cutting-edge translational studies reported that, in a mouse wound *in vivo* model, biomimetic nanocarriers targeting Engrailed-1 (*En1*) lineage-positive fibroblasts delivered siRNA to silence this core profibrotic switch, achieving scarless healing with a single administration (24). At the level of cellular rejuvenation, studies in cultured human fibroblasts directly compared an optimized chemical reprogramming protocol with *OSKM* transgenic induction, revealing that both reduced senescence markers and mitochondrial reactive oxygen species levels. However, chemical reprogramming yielded a more homogeneous cell population, did not induce pluripotency markers, and circumvented telomerase activation and acute cellular senescence stress, demonstrating superior controllability (25).

Epigenetic and metabolic reprogramming fundamentally reshapes cell function without altering the DNA sequence, acting through DNA methylation remodeling, histone modifications, and RNA epitranscriptomic regulation. Study in keratinocytes under high-glucose conditions showed that blocking DNA demethylation and promoting histone H3 methylation with epigenetic inhibitors significantly improved migration, a finding subsequently validated in streptozotocin-induced diabetic mouse skin and wound models (26). At the RNA epitranscriptomic level, recent reviews integrating multiple *in vitro* and *in vivo* studies indicate that m^6^A modifications exert spatiotemporal control over keratinocyte migration, fibroblast activation, and macrophage polarization; their dysregulation exacerbates impaired diabetic wound healing by amplifying oxidative stress and autophagy deficiency (27). On the metabolic front, *in vitro* experiments on bone marrow-derived macrophages conclusively demonstrate that hyperglycemia and mitochondrial dysfunction in the diabetic microenvironment lock macrophages into a glycolysis-dependent pro-inflammatory M1 phenotype (28). Furthermore, *in vitro* studies reveal that subjecting senescent fibroblasts within 3D collagen hydrogels to static compressive force reshapes chromatin architecture and restores cell migration via *ERK* signaling, thereby laying a preclinical foundation for the translational application of “mechano-epigenetic reprogramming” in wound healing (29).

Nanomaterial- and biomaterial-assisted reprogramming addresses the critical translational gap in the spatiotemporally precise delivery of bioactive molecules. Lipid nanoparticles (*LNPs*), a core platform for mRNA delivery, have already been clinically validated in inactivated virus vaccines (30). In a diabetic mouse wound study, reactive oxygen species (*ROS*)-responsive *LNPs* simultaneously delivered interleukin-4 (*IL-4*) mRNA and actively scavenged excess *ROS* in the wound microenvironment, promoting M1-to-M2 macrophage polarization (31). For integrated strategies, a study in diabetic rats employed an integrin αvβ3-targeted triple-targeting core–shell nanosystem to deliver miR-146a-5p to macrophages, endothelial cells, and fibroblasts, synergistically achieving immunomodulation, angiogenesis, and collagen deposition (32). Meanwhile, a temporally programmed bilayer hydrogel in a diabetic mouse model released IL-10 in the early phase and vascular endothelial growth factor (*VEGF*) and platelet-derived growth factor (*PDGF*) in the later phase, increasing wound closure to 89.7%, the M2 macrophage proportion by 42%, and neovascular density by 2.3-fold (33). The core value of nanomaterials is now extending from that of “smart delivery carriers” to “microenvironmental homeostatic modulators”—actively participating in restoring microenvironmental homeostasis by sensing signals such as ROS, pH, and enzyme activity within the wound niche.

**Figure 2:**
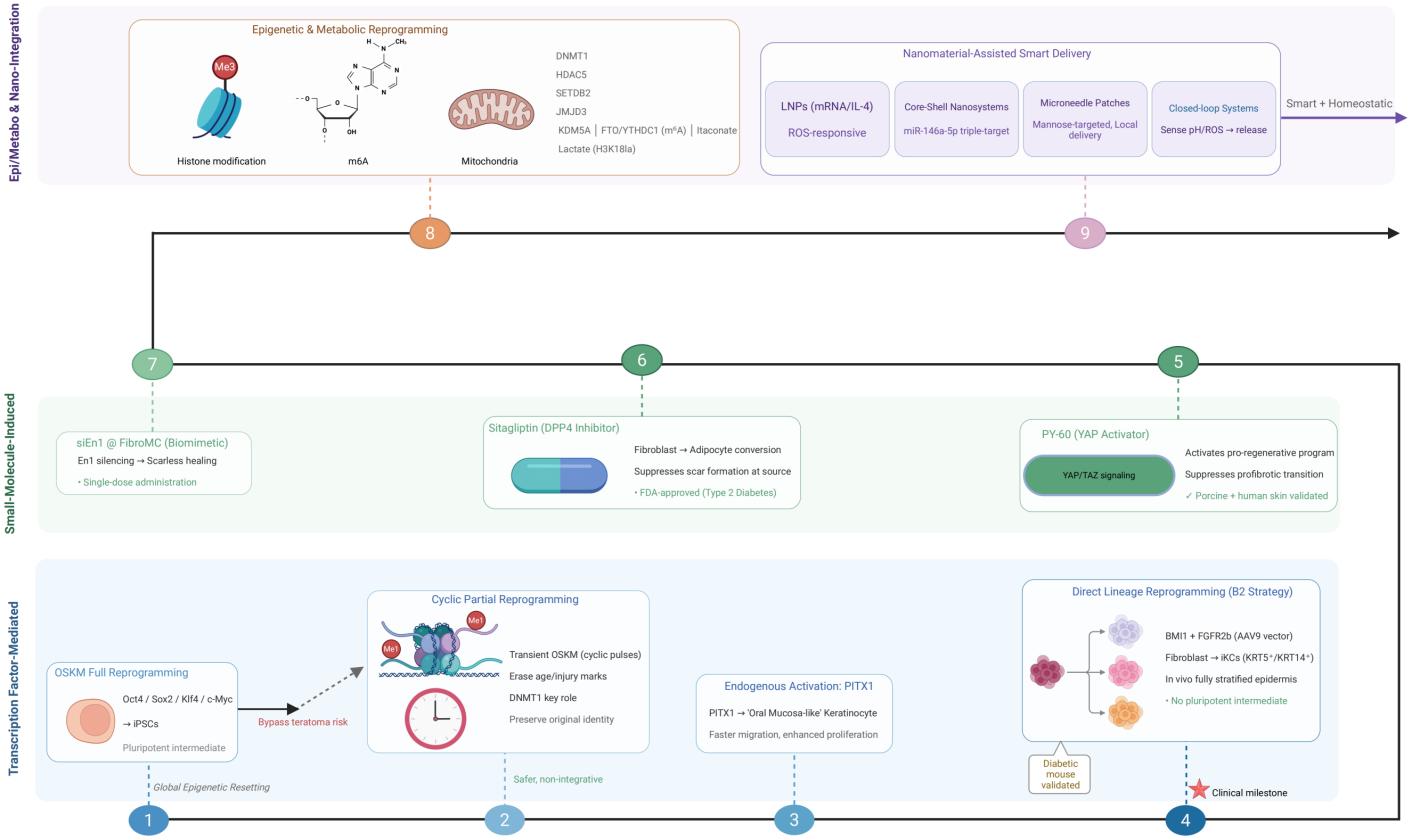
Evolution of the reprogramming toolbox. Transcription factor-based strategies progressed from OSKM-mediated full reprogramming (2006) to partial reprogramming (2016) that erases epigenetic marks without pluripotency induction, and ultimately to direct lineage reprogramming—exemplified by *in vivo* fibroblast-to-iKC conversion via BMI1+FGFR2b (B2 strategy, 2025)—which bypasses pluripotent intermediates entirely. Small-molecule approaches (PY-60, sitagliptin, siEn1@FibroMC) offer dose-controllable, integration-free alternatives with reduced safety thresholds. Epigenetic/metabolic modulation (DNMT1, HDAC5, m^6^A, H3K18la, itaconate) and nanomaterial-assisted delivery (LNPs, core-shell, microneedles) converge toward spatiotemporally precise, microenvironment-responsive ‘smart’ reprogramming. ★ denotes clinical translation milestones; arrows indicate iterative refinement toward safety and precision. (Create with BioRender).

The reprogramming toolbox for wound repair has evolved into a multi-tiered architecture spanning genetic manipulation, chemical induction, epigenetic and metabolic regulation, and nanomaterial-assisted delivery: transcription factor strategies achieve functional rejuvenation and identity conversion through partial reprogramming and direct lineage reprogramming, respectively; small-molecule strategies offer alternatives with a lower safety threshold; epigenetic and metabolic regulation unlocks the underlying codes for reversibly reshaping cell function; and nanomaterials, in turn, enable the spatiotemporally precise delivery of all these strategies within the real wound microenvironment(**Table 1**) (**Figure 2**).

**Table 1:** Comparison of the four major reprogramming technology platforms for wound repair.

Technology platform	Core principle	Representative strategies	Key advantages	Key limitations	Highest stage of clinical translation	Ref(s)
Transcription Factor-Mediated	Pioneer transcription factors are forcibly expressed to initiate epigenetic remodeling of cell identity and rewrite gene regulatory networks	Direct lineage reprogramming: BMI1+FGFR2b (fibroblast → keratinocyte-like cells); Partial reprogramming: cyclic transient OSKM expression	Fundamental conversion of cell identity; unambiguous mechanisms and robust effects	Genomic integration and tumorigenic risk with viral vectors; in vivo efficiency remains to be optimized	In vivo (mouse models)	(10, 11, 17, 18)
Small-Molecule -Induced	Cell-permeable small molecules target endogenous signaling pathways and chromatin modifiers to indirectly reconfigure the transcriptional landscape	Pro-regenerative: YAP activator PY-60; Anti-fibrotic: DPP4 inhibitor sitagliptin; Lineage silencing: En1 siRNA nanocarrier	No genomic integration risk; dose-controllable; reversible upon withdrawal; relatively low cost	Off-target effects and long-term epigenetic mutagenicity risk unknown; may require frequent administration	In vivo (porcine model) / Ex vivo (human skin)	(22–24)
Epigenetic & Metabolic Regulation	Chemical modification of DNA, histones, or RNA, or reprogramming of energy metabolism, to modulate cell function without altering the DNA sequence	Epigenetic inhibitors restore fibroblast/keratinocyte function; Metabolic modulation: itaconate, mechanical force	Precise, reversible, "corrects" rather than "rewrites"; multiple mechanistic entry points	Insufficient target specificity; systemic side-effect profile unclear	In vivo (diabetic mouse models)	(26, 29, 74, 75)
Nanomaterial- Assisted Delivery	Nano-/biomaterials achieve targeted, stimuli-responsive delivery of reprogramming factors, or actively participate in microenvironmental reprogramming	ROS-responsive LNPs delivering IL-4 mRNA; Temporally programmed bilayer hydrogel for coordinated immuno-angiogenesis	Solves the "last-mile" delivery bottleneck; integrable multimodal signals; spatiotemporally controllable	Scalable manufacturing and quality control standards immature; long-term biosafety concerns remain	In vivo (diabetic mouse/rat models)	(31–33, 84)

## 3 Fibroblasts: the identity transformation from scar makers to regenerative repairers

### 3.1 Fibroblasts in wound healing: from physiological repair executors to pathological scar makers

Fibroblasts represent the most critical structural cell population in wound healing. During physiological repair, they synthesize, deposit, and remodel the extracellular matrix (ECM) and, by differentiating into myofibroblasts, drive wound contraction; after tissue closure, they undergo timely apoptosis and regression to restore dermal homeostasis (34). In the chronic wound microenvironment, sustained hyperglycemia, mechanical signal dysregulation, and aberrant epigenetic programming drive the persistent activation of fibroblasts, leading to unbalanced ECM synthesis and degradation, and ultimately stalled repair or pathological scarring (35). In pathological healing, fibroblasts remain persistently activated and deposit excessive abnormal ECM, leading to loss of appendages, tissue stiffness, and pathological scar formation (36). Therefore, the precise modulation of fibroblast phenotype—rather than their outright elimination—constitutes the central issue in reprogramming-based intervention (**Table 2**).

**Table 2:** Key fibroblast subpopulations in skin and wounds: markers, functional characteristics, spatiotemporal distribution, and reprogramming targets.

Subset classification		Specific markers	Functional characteristics in wound healing	Spatiotemporal distribution in wounds	Reprogramming targets	Ref(s)
Pro-regenerative Subsets	En1-lineage-negative fibroblasts (ENFs)	En1^-^; CD26(Dpp4)^+^; Pdgfra^+^	Pro-regenerative; deposit a fibronectin-rich provisional matrix; activate Wnt/Trps1 pathways	~25% of adult dermal fibroblasts; participate in early repair	Prevent postnatal En1 activation to maintain pro-regenerative phenotype	(24, 52)
	Prg4^+^ fibroblasts	Prg4^+^	Appear transiently at wound edge early after injury; contribute to early repair microenvironment; replaced by other subsets within days	Early wound edge (transient)	Signaling dynamics under active investigation	(38)
	Col25a1^+^ fibroblasts	Col25a1^+^	Long-resident; participate in ordered ECM deposition; signal crosstalk with immune cells in the sub-epidermal region	Sub-epidermal region (late wound healing to scar stage)	Key node in signaling interaction networks	(38)
Pro-fibrotic Subsets	En1-lineage-positive fibroblasts (EPFs)	En1^+^	Pro-fibrotic; deposit dense, parallel-aligned type I collagen fibers; constitute the main structural basis of scar tissue	~75% of adult dermal fibroblasts; upon injury, ENFs can convert into EPFs through postnatal En1 activation	Biomimetic fibroblast nanocarrier (FibroMC) delivering siEn1 silences the pro-fibrotic program; single-dose administration achieves scarless healing	(24, 52)
	Pamr1^+^ fibroblasts	Pamr1^+^	Long-resident; localized to deep scar regions; signal crosstalk with immune cells	Deep scar region (late healing to scar maturation)	Key node in signaling interaction networks	(38)
	Myofibroblasts	α-SMA (Acta2)^+^; Myl9^+^; Tagln^+^	Contract wound; massively synthesize ECM; in physiological healing they undergo timely apoptosis and regression; in pathological states they persist causing fibrosis	Wound granulation tissue (proliferative phase); persist in pathological scars	Revert into adipocytes via BMP signaling; mechanical signal inhibition induces apoptosis/regression	(34, 53)

### 3.2 Molecular basis of fibroblast dysfunction: heterogeneity, mechanotransduction and epigenetic memory

Single-cell transcriptomics has fundamentally overturned the traditional view of dermal fibroblasts as a functionally homogeneous population (37). **Table 2** summarizes the recognized fibroblast subpopulations, their markers, functional characteristics, spatiotemporal distribution within wounds, and reprogramming targets, systematically capturing their distinct roles in wound repair.

Integrative single-cell analysis by Almet et al. further revealed their spatiotemporal dynamics: Prg4-expressing fibroblasts emerge transiently along the wound edge early after injury and are replaced within days by long-residing subpopulations such as *Col25a1^+^* (subepidermal) and *Pamr1^+^* (deep scar), with almost no interconversion between them, indicating that differentiation trajectories are highly regionally and temporally constrained (38). Liu et al., analyzing scRNA-seq data from 89,148 cells, identified 11 myofibroblast subclusters, among which specific subsets were significantly enriched in ulcer tissue and involved in bacterial response and angiogenesis (39).

Building upon this heterogeneity, aberrant mechanotransduction serves as a critical upstream trigger of the profibrotic fibroblast phenotype and is deeply intertwined with downstream epigenetic programming, together forming a pathogenic loop of “mechanosensing → epigenetic remodeling → fibrotic epigenetic memory lock-in” (40).

Fibroblasts sense mechanical changes in the ECM through the evolutionarily conserved mechanosensitive cation channel *Piezo1*. Upon membrane stretch caused by increased tissue tension or elevated ECM stiffness in the wound bed, *Piezo1* opens and mediates a rapid influx of Ca²^+^ (118). The rise in intracellular Ca²^+^ activates the *RhoA/ROCK* signaling cascade, which promotes actin stress fibre assembly and concurrently inhibits the Hippo pathway kinases *LATS1/2. LATS1/2* inhibition reduces phosphorylation of the transcriptional co-activators *YAP* and *TAZ*, preventing their cytoplasmic retention and allowing massive nuclear translocation (119, 120). Inside the nucleus, *YAP/TAZ* associate predominantly with *TEAD* family transcription factors to drive the expression of a battery of pro-fibrotic target genes, including α-smooth muscle actin, collagen type I (*COL1A1, COL1A2*), and connective tissue growth factor (121). Thus, the *Piezo-YAP* axis constitutes a complete signalling chain from “mechanical force sensation” to “pro-fibrotic transcriptional programming,” directly converting physical cues from the wound microenvironment into fibroblast phenotypic output.

Mature adipocytes can transdifferentiate into profibrotic fibroblasts via *Piezo1/Piezo2*-mediated mechanosensing, and approximately 10% of wound fibroblasts at day 14 post-injury originate from this source (41). Multiomics studies demonstrate that inhibiting Piezo1 not only prevents scar formation upon wounding, but that a local intradermal injection can even markedly alleviate established scars at 120 days post-injury—evidenced by the reappearance of hair follicles and other appendages and the restoration of normal ECM architecture (42). Spatial proteomics and transcriptomics further revealed that mechanically sensitive fibroblast subpopulations were significantly reduced in scars receiving Piezo1 inhibitor injection, indicating that sustained *Piezo1* mechanical signaling plays a critical role in maintaining dermal fibrosis (43). In parallel, a single application of the *YAP* inhibitor verteporfin to wounds in a porcine model blocked scar formation and drove skin regeneration; scRNA-seq analysis revealed that its mechanism involves reducing fibrosis-associated fibroblast subsets, enriching pro-regenerative fibroblasts, and upregulating *IL-33* expression (44).

*Piezo1/YAP* mechanical signals extend beyond cytosolic biochemical cascades: they relay mechanical information to the nucleus through specific molecular pathways, encoding it as profibrotic epigenetic memory at the chromatin level and thereby “permanently locking” fibroblasts in a pathogenic state (45). In the early phase of acute wound healing, moderate activation of *Piezo-YAP* is beneficial: it drives fibroblast-to-myofibroblast differentiation, facilitates wound contraction, and promotes ECM deposition to accelerate wound closure (22). However, in chronic non-healing wounds and hypertrophic scars this pathway becomes persistently and aberrantly activated, locking fibroblasts in a hyper-contractile, ECM-overproducing pathological state that classifies them as “scar makers.” In diabetic ulcers and other chronic wounds, sustained mechanical tension, abnormally cross-linked ECM, and inflammatory signals combine into a positive-feedback loop that keeps Piezo1 open and *YAP/TAZ* constitutively nuclear, driving an irreversible fibrotic programme. Therefore, the *Piezo-YAP* pathway represents both a pivotal mechanistic node underlying fibroblast identity dysfunction and a highly promising interventional target for fibroblast reprogramming strategies. Pharmacological inhibition of *Piezo1* or disruption of *YAP–TEAD* interaction holds the potential to release fibroblasts from the pro-fibrotic lock and restore their regenerative competence^[126]^.

**Figure 3:**
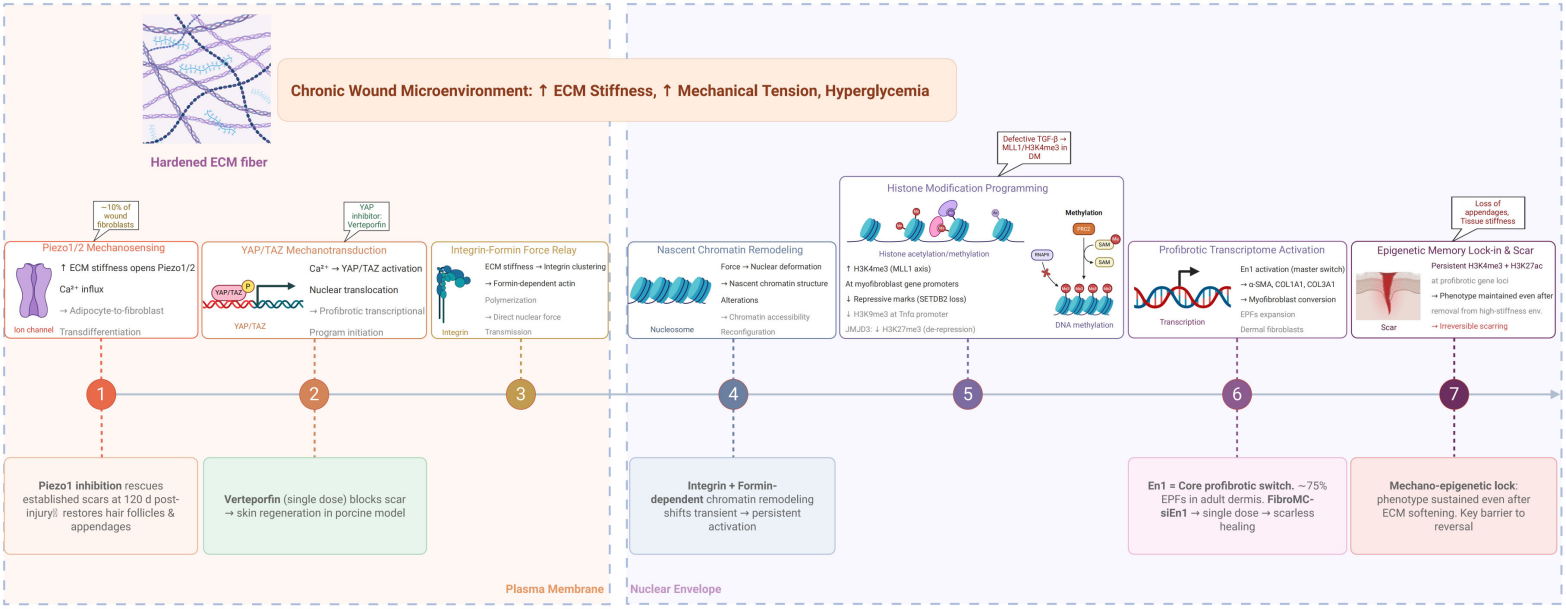
Schematic of the mechano-epigenetic lock-in driving fibroblast-to-myofibroblast conversion and pathological scarring. (1).Piezo1/2 channels sense elevated ECM stiffness in the chronic wound microenvironment. (2).Calcium influx activates YAP/TAZ, promoting nuclear translocation. (3).Integrin clustering and formin-dependent actin polymerization relay mechanical force directly to the nucleus. (4).Nuclear force alters nascent chromatin structure, shifting fibroblasts from transient to persistent activation. (5).Histone modifications are reprogrammed—MLL1-mediated H3K4me3 increases at myofibroblast gene promoters, while repressive marks (H3K9me3) are lost. (6).The transcription factor En1 acts as a master profibrotic switch, driving EPF expansion (∼75% of adult dermal fibroblasts) and activating α-SMA/COL1A1. (7).Persistent H3K4me3/H3K27ac deposition establishes an epigenetic memory lock, sustaining the profibrotic phenotype even after ECM softening and leading to irreversible scarring. Key interventional targets: Piezo1 inhibition, YAP blockade (Verteporfin), and En1 silencing (FibroMC-siEn1). (Create with BioRender).

A landmark review has pointed out that sustained mechanical tension, via direct force transmission to the nucleus, evokes durable myofibroblast epigenetic memory—encompassing key modifications such as DNA methylation and histone acetylation/methylation—that sustains the profibrotic phenotype even after withdrawal from a high-stiffness environment (46). ECM stiffness mediates this chromatin remodeling through integrin- and formin-dependent mechanisms, shifting fibroblasts from “transient activation” to “sustained activation” (47). Moreover, another study further confirms that alterations in nascent chromatin structure directly govern the activation of the profibrotic transcriptome and promote the emergence of myofibroblasts in organ fibrosis, delineating the complete signaling chain of mechanical signal → chromatin remodeling → profibrotic gene activation (48). Integrating these cutting-edge lines of evidence, a clear interlinked mechanistic chain emerges: Piezo1 senses ECM stiffness changes, activates the *YAP/TAZ* mechanotransduction axis, and relays force signals to the nucleus via integrin-formin-dependent pathways. This in turn regulates nascent chromatin architecture and dynamic histone modifications, activates the profibrotic transcriptional program, and drives fibroblast-to-myofibroblast conversion with the establishment of persistent epigenetic memory—ultimately leading to long-term maintenance of the profibrotic phenotype and irreversible pathological scarring, even after cells are removed from the high-stiffness environment (49)(**Figure 3**).

Canonical *TGF-β/SMAD* signaling acts as a master transcriptional driver of the Engrailed-1 (*En1*)-positive profibrotic fibroblast (*EFP*) lineage. Sustained *TGF-β1* stimulation, in concert with *YAP/TAZ* mechanosignaling, directly binds to the *EN1* enhancer region through *SMAD2/3* and *TEAD* complexes, transcriptionally activating *En1* expression and locking fibroblasts into an *EPF* identity characterized by high expression of *α−SMA* and type I collagen (122). Once established, this *EPF* state is epigenetically reinforced by persistent deposition of active histone marks (*H3K4me3* and *H3K27ac*) at profibrotic gene loci, as described earlier in the mechano-epigenetic chain.

At the epigenetic level, in diabetic wounds, the *TGF-β*-driven programming that increases H3K4me3 at the promoters of myofibroblast marker genes via the *MLL1/H3K4me3* axis is markedly defective (50). *JMJD3*, an *H3K27me3* demethylase, has been identified as a key epigenetic regulator of this *TGF-β*-mediated conversion (51). More critically, the transcription factor *En1* has been established as the core molecular switch determining the profibrotic fate of fibroblasts: *En1* lineage-negative fibroblasts (*ENFs*) possess pro-regenerative properties, whereas *EPFs* exhibit a profibrotic phenotype; approximately 75% of adult dermal fibroblasts belong to the latter, which indicates that the majority of adult dermal fibroblasts are committed to the early phase of the fibrotic repair response, whereas only a subset-approximately 25%-of EN1-negative fibroblasts retain regenerative capacity, enabling scar-free wound healing, *de novo* hair follicle neogenesis, and restoration of native skin architecture. Upon injury, *ENFs* can convert into *EPFs* through postnatal *En1* activation. Building on this principle, a biomimetic fibroblast nanocarrier (*FibroMC*) delivering a single dose of *siEn1* restored normal collagen architecture, regenerated skin appendages, and prevented scar formation (24, 52).

### 3.3 Redirecting fibroblast lineage commitment: identity and functional reprogramming for scarless wound repair

At the level of fate conversion, multiple strategies have demonstrated that the profibrotic fate of fibroblasts can be redirected, yet they differ markedly in their technical routes, efficiency, and safety profiles.

#### 3.3.1 Transcription factor-mediated transdifferentiation

In dedifferentiation reprogramming, Plikus et al. demonstrated that myofibroblasts can re-express adipogenic transcription factors and convert into adipocytes in response to *BMP* signals from newly formed hair follicles, opening a novel avenue for “regenerating adipocytes from scar tissue” (53). In cross-germ-layer direct reprogramming, *in situ* delivery of *BMI1* and F*GFR2b* via *AAV9* vectors directly converts fibroblasts in diabetic mouse wounds into keratinocyte-like cells. This strategy exhibited clear reprogramming efficiency in L929 mouse fibroblasts *in vitro* and, in db/db diabetic mouse wounds *in vivo*, markedly accelerated wound closure, reconstructed stratified epidermis, restored barrier function, and significantly reduced animal mortality. Its core advantage lies in completely bypassing the pluripotent stem cell stage and cell transplantation procedures (10).

#### 3.3.2 Small-molecule strategies

The DPP4 inhibitor sitagliptin directs fibroblast-to-adipocyte conversion, suppressing scar formation at its source—a finding validated by cell-based and *in vivo* mouse wound studies (23, 54). Its key translational advantage is that, as an already approved drug for type 2 diabetes, sitagliptin is backed by extensive clinical safety and pharmacokinetic data; pursuing this drug-repurposing route can substantially shorten the timeline to clinical access.

#### 3.3.3 Gene-free mechanical reprogramming strategies

Roy et al. cultured aged human dermal fibroblasts on micropatterned substrates to impose geometric constraints, which yielded partially reprogrammed cells with rejuvenated features (55). When implanted into *in vitro* aged skin models, these cells showed enhanced expression of ECM proteins and improved tissue regeneration. Because this strategy avoids the telomerase activation and oncogenic risks associated with OSKM transgene induction, it offers a safer route for clinical autologous transplantation.

### 3.4 Translational roadblocks of fibroblast reprogramming: unresolved questions and technical limitations

The central challenge in fibroblast reprogramming lies in achieving spatiotemporal precision:

(1) Early myofibroblast activation is essential for wound contraction; premature suppression may prevent wound closure, making the identification of the optimal therapeutic window for anti-fibrotic intervention a critical question; (2) The heterogeneity of profibrotic fibroblast subpopulations across different wound types (acute burns, diabetic ulcers, hypertrophic scars) has yet to be systematically characterized, hampering the development of individualized strategies; (3) The coordinated integration of mechanical signaling inhibitors (e.g., verteporfin), nanodelivery systems (*FibroMC*), and direct reprogramming factors (the B2 combination, *BMI1* and *FGFR2b*) entails complex optimization of temporal sequence and dosing, for which systematic preclinical data remain lacking (56).

Fibroblast reprogramming not only reverses dermal fibrosis, but also creates a permissive microenvironment for keratinocyte re-epithelialization and macrophage phenotypic switching, forming the structural basis for coordinated wound repair.

## 4 Keratinocytes: activating the regeneration engine to reconstruct the epidermal barrier

### 4.1 Keratinocytes in wound repair: epidermal barrier builders and re-epithelialization core executors

Keratinocytes constitute over 95% of epidermal cells and serve as the central executors of the skin’s physical barrier and post-injury re-epithelialization (57). During physiological healing, keratinocytes at the wound edge undergo a precisely timed cascade of activation, migration, proliferation, and differentiation, progressively covering the exposed wound surface and re-establishing the stratified epidermal architecture. In chronic wounds, however, the aforementioned negative conditions profoundly suppress their migratory and proliferative capacity, impair autophagy, and increase apoptosis, stalling re-epithelialization, leaving the wound persistently open, and markedly elevating infection risk. Conventional strategies rely on autologous epidermal cell grafts or stem-cell-derived supplementation, yet face translational hurdles including donor-site morbidity, low expansion efficiency, and immune rejection.

To address these obstacles, two strategies with distinctly different clinical application scenarios have emerged in keratinocyte reprogramming. The first focuses on “endogenous activation”—using the transcription factor *PITX1* to awaken the regenerative potential of skin-resident keratinocytes, endowing them with rapid-healing characteristics resembling oral mucosa. This strategy is suited for small- to medium-sized wounds where a certain number of normal keratinocytes remain at the wound edge but are functionally suppressed by metabolic disturbances (20). The second focuses on “exogenous generation”—employing the *BMI1* and *FGFR2b* factor combination (B2) to directly transdifferentiate abundant local fibroblasts in the wound into induced keratinocyte-like cells (iKCs). This strategy is indicated for chronic wounds with large-area full-thickness skin defects where the wound edge lacks a source of normal epidermal cells, such as diabetic foot ulcers. These two strategies complement each other, together forming a complete clinical-resolution framework for keratinocyte reprogramming (10).

### 4.2 Molecular mechanisms of keratinocyte dysfunction: epigenetic remodeling and metabolic reprogramming

The molecular basis for reprogramming intervention lies in a deep dissection of the epigenetic and metabolic regulatory networks governing keratinocyte function. At the level of transcriptional identity, Overmiller et al., using single-cell RNA sequencing and spatial transcriptomics, found that the transcription factor *PITX1* is highly expressed in oral mucosal epithelium but barely detectable in skin keratinocytes, uncovering the molecular basis for the differential healing capacity between skin and oral mucosa (20).

At the DNA methylation level, promoter methylation plays a critical regulatory role in keratinocyte differentiation and dysfunction. Ding et al. showed that hypermethylation of the miR-125b-5p and miR-199b-5p promoters transcriptionally silences these miRNAs (58). Under normal conditions, both miRNAs post-transcriptionally repress *ΔNp63*; their loss therefore de-represses *ΔNp63*, a master regulator of epidermal stratification. Supraphysiological *ΔNp63* aberrantly activates *PI3K/AKT/mTOR* signaling, driving keratinocytes into a terminally differentiated state that, in chronic wounds, locks cells in a hyperproliferative yet non-migratory phenotype and blocks re-epithelialization (58). Restoring miR-125b-5p/199b-5p or pharmacologically inhibiting *PI3K/AKT/mTOR* reverses this differentiation block and restores motility, identifying this axis as a high-value reprogramming target (58). More broadly, global DNA methylation status also modulates keratinocyte behavior: compared with plastic or collagen I substrates, glass-matrix culture induces genome-wide hypomethylation, which is accompanied by faster proliferation, upregulation of differentiation markers, and markedly enhanced migration, further underscoring the epigenetic coupling to repair function (59).

At the histone modification level, several landmark studies have broadened our understanding of keratinocyte epigenetic regulation from multiple dimensions. Zhang et al. uncovered a novel non-histone deacetylation mechanism: in wound-edge keratinocytes, histone deacetylase 5(*HDAC5*) transiently translocates from the nucleus to the cytoplasm, where it deacetylates α-actinin-4 (*ACTN4*) at lysine 417 (*K417*), enabling *ACTN4* to enter the nucleus and act as a transcriptional co-activator that cooperatively upregulates cystatin A expression via *YBX1* to promote re-epithelialization (60). An *HDAC5*-selective activator, *G194-0712*, developed based on this mechanism, effectively accelerated wound closure in three mouse chronic wound models—diabetic, ischemic, and radiation injury (60). In parallel, Mangum et al., using diet-induced obese and db/db mouse models of type 2 diabetes together with wound tissues from T2D patients, performed single-cell sequencing and found that the repressive histone methyltransferase *SETDB2* is markedly reduced in diabetic wound keratinocytes. Chromatin immunoprecipitation confirmed that loss of *SETDB2* erases the repressive *H3K9me3* mark at the *Tnfα* promoter, driving overexpression of inflammatory genes; the *IFNβ/JAK/STAT* signaling axis was identified as a key upstream regulatory pathway of *SETDB2 (*61). Moon et al. further demonstrated that elevated IL−17A in diabetic wounds induces the histone demethylase *JMJD3* via the *TRAF6/NFκB* pathway; *JMJD3* then erases repressive *H3K27me3* marks at the promoters of anti-migratory genes (*Itga3, Timp1*) and inflammatory genes (*Ccl20, Cxcl1*, etc.), leading to impaired keratinocyte migration and intensified inflammation. Keratinocyte-specific deletion of *IL−17A* signaling or *JMJD3* both improved wound healing in diabetic mice (62). Together, these three studies—addressing histone deacetylation (*HDAC5–ACTN4* axis), histone methylation (*SETDB2–H3K9me3* axis), and histone demethylation (*JMJD3–H3K27me3* axis)—construct a multidimensional mechanistic map of histone modification dysregulation in chronic wound keratinocytes.

At the RNA epitranscriptomic level, m^6^A modification has been shown to play a central regulatory role in keratinocyte autophagy. The m^6^A demethylase *FTO* is markedly downregulated in diabetic epidermis and regulates autophagy by demethylating the 3′ UTR of *TRIB3* mRNA to enhance its *YTHDF2*-dependent stability (63). The m^6^A reader *YTHDC1* is similarly reduced in diabetic keratinocytes, where it directly binds *SQSTM1/p62* mRNA to modulate its stability; knockdown of either Ythdc1 or Sqstm1 suppresses epidermal autophagy and delays wound healing (63). Together, from the vantage points of m^6^A erasure and m^6^A readout, *FTO* and *YTHDC1* uncover a core mechanism through which chronic hyperglycemia disrupts the m^6^A–RNA–autophagy axis to drive keratinocyte dysfunction.

A deeper regulatory layer lies in the cascade integration of the metabolism–epigenetics–transcription axis. Hu et al. demonstrated that *METTL3* promotes keratinocyte proliferation by enhancing m^6^A modification of *DNMT1* mRNA, and that elevated lactate in wounds activates *METTL3* transcription through inducing histone H3K18 lactylation, thus delineating a complete regulatory chain: hyperglycemia→lactate accumulation→*H3K18* lactylation→*METTL3* activation→*DNMT1* m^6^A modification→keratinocyte proliferation (64).

**Figure 4:**
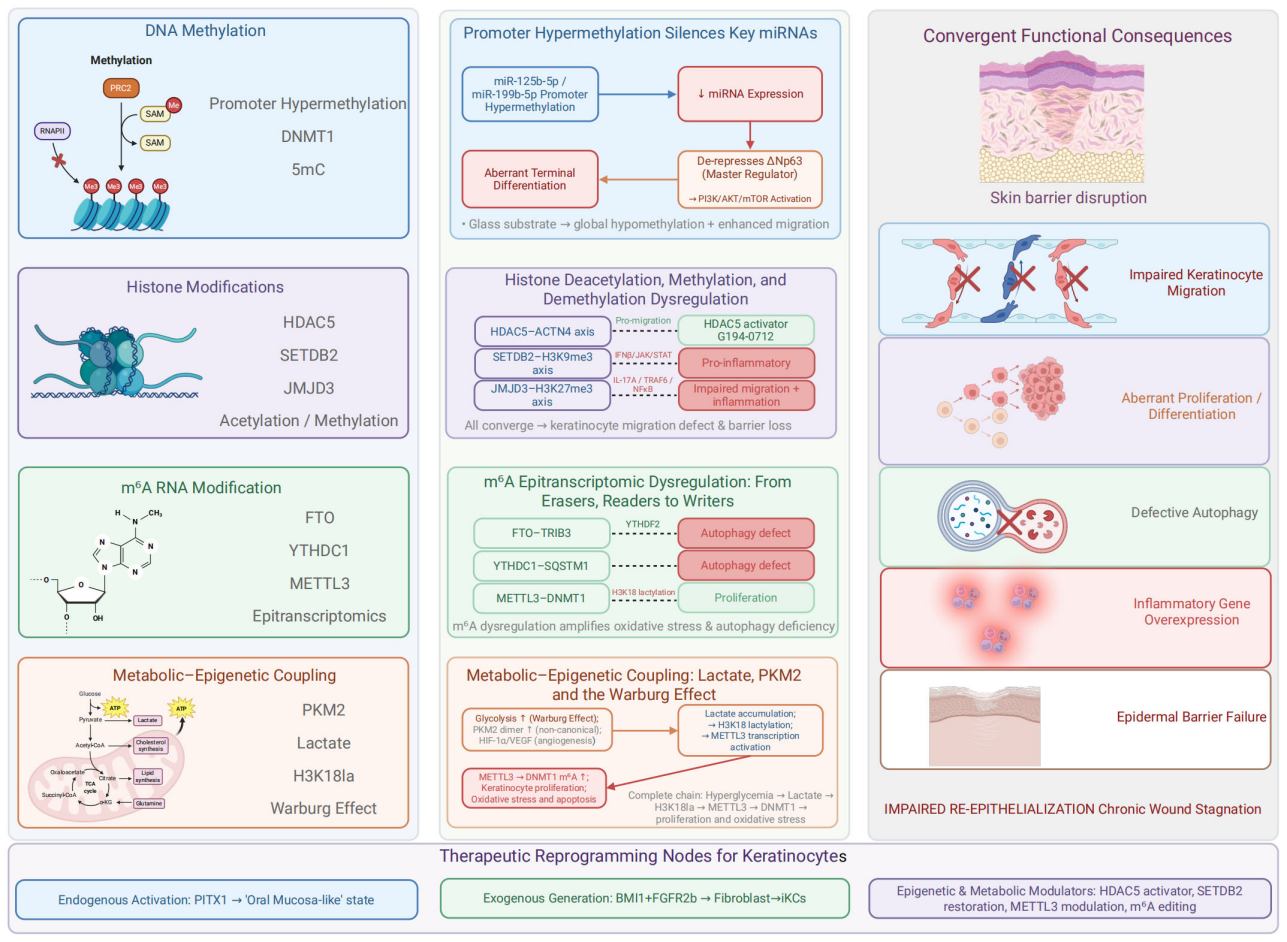
Multi-dimensional molecular dysregulation in chronic wound keratinocytes. DNA Methylation: Promoter hypermethylation silences miR-125b-5p/199b-5p, de-repressing ΔNp63 and aberrantly activating PI3K/AKT/mTOR-driven differentiation. Histone Modifications: HDAC5 deacetylates ACTN4 to promote CSTA-mediated migration; SETDB2 loss reduces H3K9me3 at the Tnfα promoter, driving inflammation; and IL-17A–induced JMJD3 erases H3K27me3 at anti-migratory and inflammatory gene loci. m⁶A RNA Modification: FTO and YTHDC1 downregulation impairs autophagy via TRIB3 and SQSTM1/p62 mRNA; METTL3 (activated by H3K18 lactylation) enhances DNMT1 m⁶A to push proliferation. Metabolic–Epigenetic Coupling: Glycolytic PKM2 dimers promote VEGF/HIF-1α, while lactate-driven H3K18la activates METTL3, linking hyperglycemia to epigenetic remodeling. These pathways collectively result in impaired re-epithelialization and barrier failure, amenable to reprogramming via PITX1 activation, B2 transdifferentiation, and epigenetic modulators. (Create with BioRender).

At the level of metabolic programming, wound keratinocytes exhibit a “Warburg effect” akin to tumor cells—preferentially utilizing glycolysis over oxidative phosphorylation even under aerobic conditions (65). Recent studies reveal that pyruvate kinase M2 (*PKM2*) is markedly upregulated in wound-edge keratinocytes from day 3 post-injury, peaks at day 5, and remains highly expressed until wound closure. Notably, *PKM2* in wounds exists almost entirely as the metabolically inactive dimer, indicating it primarily performs a non-canonical “moonlighting” function: dimeric *PKM2* promotes *VEGF* expression in a HIF-1α-dependent manner, directly coupling the glycolytic metabolic reprogramming of keratinocytes to wound angiogenesis (**Figure 4**). This metabolic adaptation ensures rapid energy supply for proliferating keratinocytes while concurrently creating conditions for granulation tissue formation through pro-angiogenic signaling (66).

### 4.3 Restoring keratinocyte regenerative capacity: endogenous phenotype activation and *in situ* lineage reprogramming

For endogenous activation of regenerative potential, ectopic expression of *PITX1* reprograms skin keratinocytes into a “quasi-oral keratinocyte” state—characterized by faster migration and enhanced proliferation, while also remodeling intercellular communication networks and establishing a favorable inflammatory microenvironment (20). The key insight from this strategy is that the inherent rapid-healing capacity of oral mucosa can be extracted and “transplanted” into skin, and that a single master transcription factor is sufficient to initiate a transcriptional program that overturns cell identity and function (116). Its typical application is in small- to medium-sized wounds where normal keratinocytes are still present at the wound edge but are functionally suppressed by hyperglycemia or chronic inflammation—here, merely “awakening” the regenerative potential of the resident cells can accelerate re-epithelialization (117).

For *in situ* fibroblast conversion, *AAV9* vector-mediated *in situ* delivery of the dual-factor combination *BMI1* and *FGFR2b* (B2) directly reprograms wound fibroblasts into iKCs expressing markers such as *KRT5* and *KRT14*, which then reconstruct a fully stratified epidermal architecture and restore barrier function (10). Transcriptomic analysis confirmed that iKCs not only closely resemble normal keratinocytes morphologically, but also display a molecular expression profile converging toward that of native keratinocytes (67). This strategy completely bypasses the pluripotent stem cell intermediate and exogenous cell transplantation. Its typical application is in large-area full-thickness skin defects, such as diabetic foot ulcers, where the wound edge lacks a source of normal epidermal cells and the epidermis must be “regenerated” through cross-germ-layer transdifferentiation of fibroblasts.

### 4.4 Clinical translation hurdles of keratinocyte reprogramming: safety, efficiency and controllability

Despite the impressive preclinical efficacy demonstrated by these strategies, the translation of keratinocyte reprogramming still faces several core bottlenecks that must be systematically addressed before advancing to clinical trials.

First, the functional integrity and long-term safety of transdifferentiated iKCs. An *in vitro* study evaluating the barrier function of iPSC-derived keratinocytes showed that, when cultured on collagen gels derived from human primary fibroblasts, transepidermal water loss decreased while the cumulative permeation of benzoic acid and isosorbide dinitrate gradually increased, indicating partial restoration of barrier function in the reconstructed epidermis. However, this iPSC-based study also suggests that the degree of barrier restoration has not yet reached the level of native epidermis (68). For iKCs generated by the B2 strategy, although they have been shown to reconstruct stratified epidermis, restore barrier function, and significantly reduce mortality in db/db diabetic mice, key evidence is still lacking: (1) whether iKCs possess the self-renewal capacity of epidermal stem cells, such as hair follicle stem cells, to maintain long-term epidermal homeostasis; (2) whether iKCs can undergo normal turnover or maintain genomic stability after wound closure, and their long-term tumorigenic risk—particularly given the dual role of *BMI1* as a *Polycomb* family member in both stem cell self-renewal and tumorigenesis—requires longer follow-up assessment in large animal models.

Second, the severe attenuation of reprogramming efficiency in the chronic wound microenvironment. The conversion efficiency of fibroblasts to iKCs is relatively controllable *in vitro*, yet it declines markedly within the complex microenvironment of diabetic wounds, interwoven with hyperglycemia, oxidative stress, and persistent inflammation. How to maintain *in vivo* reprogramming efficiency by optimizing reprogramming factor combinations, improving delivery vectors, or combining microenvironmental modulation (e.g., *ROS* scavenging, inflammation suppression) is central to the clinical feasibility of this strategy.

Third, the spatial controllability of *PITX1* reprogramming effects. The core challenge for the endogenous *PITX1* activation strategy lies in precisely confining the reprogramming effect to the wound area and switching it off upon repair completion. *PITX1*-driven excessive keratinocyte proliferation may carry the risk of epidermal hyperplasia or even tumorigenesis, and a rationally designed reversible molecular switch is still lacking.

Fourth, chronic wound-specific combined epigenetic and metabolic intervention regimens have yet to be established. Although strategies such as *HDAC5* activation, *SETDB2* restoration, *JMJD3* inhibition, miRNA promoter demethylation, and *METTL3* lactylation modulation each hold potential, the integrated effects of multiple metabolic disturbances in chronic wounds (hyperglycemia, oxidative stress, aberrant lactate) on these epigenetic targets have not been systematically elucidated. Future research needs to integrate single-cell multi-omics technologies to construct dynamic atlases of the epigenetic-metabolic state of keratinocytes across different wound regions and time windows, and to design individualized, temporally sequenced combinatorial intervention protocols accordingly.

Keratinocyte reprogramming is evolving from the proof-of-concept stage toward precision engineering. The clear distinction in application scenarios between the *PITX1* and B2 strategies, the comprehensive dissection of DNA methylation and histone modification mechanisms, and the in-depth elucidation of the metabolism–epigenetics–transcription regulatory network collectively provides a networked foundation for next-generation precision reprogramming. With the validation of the *HDAC5* activator *G194–0712* in diabetic mice, the translational window for this field is now opening (60).

## 5 Macrophages: identity remodeling from inflammatory storm to repair homeostasis

### 5.1 Macrophages in wound healing: innate immune regulators of inflammation and repair

Macrophages exert a precisely regulated biphasic function in wound healing. In acute physiological repair, infiltrating monocyte-derived macrophages initially adopt a pro-inflammatory M1 phenotype, responsible for phagocytosing bacteria and necrotic debris; under appropriate signals, they then switch in a timely manner to an anti-inflammatory, reparative M2 phenotype that drives angiogenesis, ECM deposition, and tissue remodeling (69).

The functional differences between the M1 and M2 phenotypes stem from their fundamentally distinct core metabolic features. A systematic review clearly states that M1 macrophages rely predominantly on aerobic glycolysis and the pentose phosphate pathway for rapid energy supply to meet the immediate demands of bactericidal activity, whereas M2 macrophages depend mainly on fatty acid oxidation and oxidative phosphorylation for sustained, stable energy (70). The biological significance of this metabolic preference is that glycolysis, although yielding low ATP, generates ATP rapidly, and its intermediates feed the pentose phosphate pathway to support nucleotide synthesis and *NADPH* production—well suited to the bactericidal requirements of M1 macrophages; oxidative phosphorylation, in contrast, produces high and sustained ATP yields to match the long-term tissue remodeling needs of M2 macrophages.

In chronic non-healing wounds such as diabetic wounds, however, the timely M1-to-M2 transition is fundamentally arrested—hyperglycemia and mitochondrial dysfunction drive excessive glycolysis and suppress oxidative phosphorylation, metabolically locking macrophages in a pro-inflammatory state. This constitutes the core immunopathological basis of chronic inflammation and healing stagnation. Consequently, precisely modulating macrophage phenotypic polarization and metabolic status—rather than simply suppressing inflammation—has emerged as the central strategy for reprogramming the immune microenvironment (71, 72).

### 5.2 Molecular drivers of macrophage polarization arrest: metabolic reprogramming and epigenetic regulation

At the metabolic level, the TCA cycle in M1 macrophages is truncated at isocitrate dehydrogenase and succinate dehydrogenase, resulting in the accumulation of itaconate and succinate. This breakage renders the TCA cycle unable to run in its entirety, forcing the cells to rely on glycolysis for energy. In contrast, M2 macrophages maintain an intact TCA cycle and ultimately generate ATP efficiently through oxidative phosphorylation (73). Itaconate, a TCA cycle branch product generated abundantly by the mitochondrial enzyme aconitate decarboxylase, possesses anti-inflammatory potential, as it can inhibit succinate dehydrogenase to limit ROS production, block the *STING* pathway, and activate *Nrf2 (*74). Anderson et al. recently identified lysine itaconylation as a novel post-translational modification and found that the mitochondrial deacetylase *SIRT4* acts as a lysine deitaconylase that efficiently removes this modification; *SIRT4*-deficient mice exhibited markedly delayed wound healing, establishing the critical regulatory role of the itaconylation–deitaconylation dynamic equilibrium in macrophage function (75).

**Figure 5:**
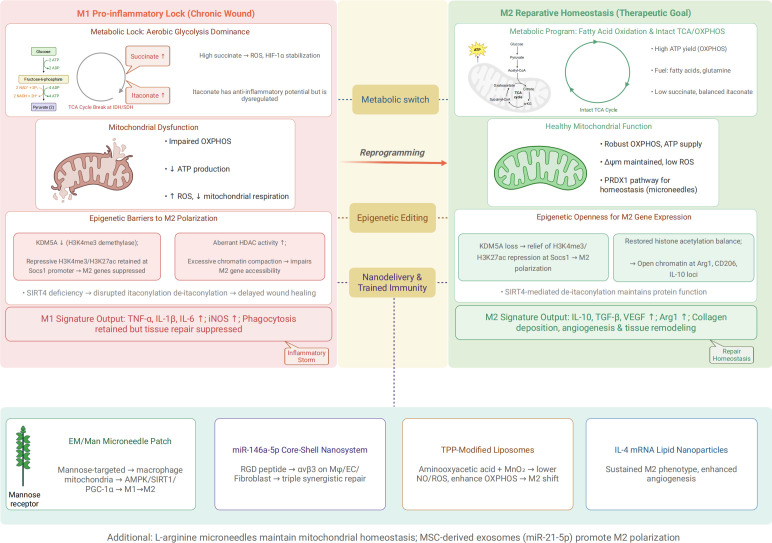
Metabolic and epigenetic polarization arrest of macrophages in chronic wounds. (Left) In the diabetic wound microenvironment, hyperglycemia and mitochondrial dysfunction drive M1 macrophages to rely on aerobic glycolysis with a broken TCA cycle, accumulating succinate and itaconate. Epigenetically, downregulation of KDM5A preserves repressive marks at M2 gene promoters, while aberrant HDAC activity compacts chromatin. (Right) The therapeutic goal is M2 polarization characterized by fatty acid oxidation, an intact TCA/OXPHOS, open chromatin at repair gene loci, and anti-inflammatory/pro-angiogenic cytokine secretion. (Center) Reprogramming strategies act at metabolic, epigenetic, and delivery levels to unlock the M1 arrest. (Bottom) Example nanoplatforms: mannose-targeted microneedles, integrin αvβ3-targeted nanoparticles, mitochondria-targeted liposomes, and IL-4 mRNA LNPs for sustained M2 programming. Trained immunity concepts are being explored for durable innate immune resetting at the hematopoietic level. (Create with BioRender).

At the epigenetic level, *KDM5A*, an *H3K4me3* demethylase, is significantly downregulated in wound macrophages and inversely correlated with M2 polarization. Knockout of *KDM5A* promotes M2 polarization by relieving the repressive *H3K4me3* and *H3K27ac* modifications at the *Socs1* promoter (76). In diabetic wounds, aberrantly elevated *HDAC* activity leads to excessive chromatin compaction, and restoring histone acetylation balance can improve the spatiotemporal distribution of macrophages (77)(**Figure 5**).

### 5.3 Trained immunity: a new frontier for macrophage functional reprogramming

The concept of “trained immunity” breaks the traditional paradigm that immune memory is exclusive to adaptive immunity—innate immune cells, upon an initial stimulus, can acquire a durable “innate immune memory” through metabolic reprogramming and epigenetic remodeling. At the mechanistic level, β-glucan-induced trained immunity shifts monocytes from oxidative phosphorylation to aerobic glycolysis, accompanied by the accumulation of fumarate and itaconate, and the persistent deposition of *H3K4me3* and *H3K27ac* at the promoters of pro-inflammatory genes, thereby establishing a long-lasting “open chromatin” memory (78, 79).

In the diabetic wound microenvironment, macrophages are driven into a persistent pro-inflammatory M1 state by a self-reinforcing metabolic–epigenetic circuit, which is further consolidated by pathological paracrine signals from dysfunctional pericytes. Hyperglycemia forces macrophages to rely on aerobic glycolysis with a broken TCA cycle, causing succinate accumulation that stabilizes *HIF-1α* and sustains *IL-1β* production (70, 73). Concurrently, accumulated succinate inhibits α-ketoglutarate-dependent histone demethylases such as *KDM5A*, thereby preserving repressive *H3K4me3* marks at M2 gene promoters and epigenetically silencing reparative gene expression (76). The metabolite itaconate, although initially providing a negative feedback brake on inflammation, paradoxically contributes to oxidative stress and mitochondrial dysfunction under chronic diabetic conditions, further perpetuating the M1 arrest (74). This intracellular lock is amplified by pericyte-derived pro-inflammatory mediators—including *CCL2*, *TNF-α*, and *IL-6*—which activate NF-κB and STAT3 signaling cascades that reinforce M1 commitment and suppress M2-associated markers such as CD206 and Arg-1 (69). It was against this background that Lv et al. dissected, for the first time, a complete “phenomenon → mechanism → intervention” chain of trained immunity in chronic wounds. Using a *Cspg4-CreERT2/+; Mat2a^(flox/flox)* mouse wound model and single-cell sequencing, they found that methionine adenosyltransferase *MAT2A* was markedly downregulated in wound pericytes and negatively correlated with the degree of inflammatory macrophage infiltration (80).

Mechanistically, *MAT2A* deficiency in pericytes impairs the recruitment of the deubiquitinase *OTUB1* to *HMGCS1*, thereby reducing *HMGCS1* protein levels, disrupting coenzyme Q synthesis, impairing mitochondrial function, and inducing pericyte senescence (80). Senescent pericytes then impose “inflammatory immune training” on infiltrating macrophages through two parallel routes: (i) secretion of a robust senescence-associated secretory phenotype (SASP) rich in *TNF-α*, *IL-6*, and *CCL2*, which continuously engages macrophage *NF-κB* and *STAT3* signaling to epigenetically reinforce M1 commitment, and (ii) intercellular transfer of dysfunctional mitochondria, which directly compromises macrophage oxidative phosphorylation, increases glycolytic dependence, and generates a metabolic milieu—characterized by elevated succinate and reduced α-ketoglutarate—that stabilizes the repressive epigenetic landscape at M2 gene loci via inhibition of *KDM5A* and aberrant *HDAC* activity (76, 77). This pericyte–macrophage crosstalk thus creates a feed-forward loop that persistently locks macrophages in a pro-inflammatory state, a concept aligned with the emerging paradigm of trained immunity in which innate immune cells acquire a durable hyperresponsive phenotype through metabolic and epigenetic reprogramming (78). Based on this mechanistic understanding, Lv et al. constructed nanoparticles loaded with self-amplifying RNA and coated with pericyte-biomimetic cell membranes, which restored stable *MAT2A* expression in senescent pericytes, effectively alleviated persistent inflammatory macrophage infiltration, and promoted wound regeneration (80). This study uncovers a cross-cellular mechanism by which non-immune resident cells (pericytes) impose “paracrine immune training” on infiltrating macrophages via metabolic–epigenetic pathways, and is the first to propose a biomimetic nanodelivery-based therapeutic strategy of “targeting senescence–interrupting training” (80).

The above research demonstrates that trained immunity can also be redirected. Delivering low-dose *TLR* agonists to bone marrow progenitors allows “benign training” that resets macrophage function at the hematopoietic source (81). It must be noted, however, that research on trained immunity in wound healing is still in its infancy overall. Only a handful of studies have directly focused on trained immunity in the chronic wound microenvironment, and key questions—including its specific pathways in different wound types, the precise metabolic–epigenetic coupling nodes, and the identification of candidate molecules for “retraining”—remain to be systematically answered.

### 5.4 Targeted nanoplatforms for precision macrophage reprogramming

The value of nanomaterials and smart biomaterials in macrophage reprogramming lies not in generic delivery functions, but in the three classes of specifically targeted delivery strategies developed to exploit the unique biology of macrophages.

The mannose receptor-targeting strategy is most representative in macrophage mitochondrial reprogramming (82). Mannose receptors are abundantly expressed on antigen-presenting cells, especially on macrophages at inflammatory sites (83). A study reported an EM/Man cascade-targeting separable core–shell microneedle patch, in which co-assembled nanoparticles of epigallocatechin gallate and metformin were modified with mannose. Through mannose–mannose receptor recognition, these nanoparticles are efficiently taken up by macrophages and subsequently accumulate in the mitochondria, activating the *AMPK/SIRT1/PGC-1α* axis to promote mitochondrial biogenesis and oxidative phosphorylation, driving M1→M2 polarization and achieving the dual goals of healing and scar inhibition in diabetic mice (84). Its core innovation lies in precisely delivering metabolic reprogramming factors to the macrophage mitochondria—the dysfunctional hub—via mannose-targeted modification.

The integrin αvβ3-targeting strategy exploits the shared expression of this receptor on macrophages, endothelial cells, and fibroblasts, enabling a single carrier to achieve triple synergistic intervention across three repair cell types (85). The miR-RPC@GelO system encapsulates a miR-146a-5p core within a lipid shell modified with Arginine–Glycine–Aspartic acid (*RGD)* and phosphatidylserine: the *RGD* peptide mediates αvβ3-specific recognition, while phosphatidylserine mimics apoptotic signals to enhance macrophage phagocytic uptake. This enables miR-146a-5p to promote M2 polarization in macrophages, restore angiogenic capacity in endothelial cells, and stimulate proliferation and collagen secretion in fibroblasts (32).

Mitochondria-targeted delivery strategies exploit the mechanistic basis of macrophage M1/M2 metabolic polarization—the difference in mitochondrial functional integrity: M1 macrophages suffer from impaired oxidative phosphorylation due to a truncated TCA cycle, whereas M2 macrophages depend on intact mitochondrial oxidative phosphorylation (86). A recently reported strategy employs triphenylphosphonium-modified mitochondria-targeted liposomes to deliver aminooxyacetic acid and hollow mesoporous manganese dioxide; after macrophage phagocytosis, this reduces *NO* and *ROS* levels, enhances mitochondrial respiration, shifts the metabolic program from aerobic glycolysis to oxidative phosphorylation, and drives M1→M2 polarization in diabetic mouse wounds (87). Meanwhile, L-arginine-loaded microneedle patches maintain mitochondrial homeostasis via the *PRDX1* pathway, restore mitochondrial membrane potential and ATP production, suppress M1 polarization while promoting M2 polarization, and accelerate wound closure in diabetic mouse models (88).

The IL-4 mRNA lipid nanoparticle “immune training” strategy exploits a macrophage-specific functional enhancement pathway. An optimized *LNP* formulation efficiently delivers *IL-4* mRNA to macrophages, stably maintaining the reparative M2 phenotype for at least one week, while simultaneously promoting M2 polarization of endogenous macrophages and enhancing angiogenesis in inflammatory injury models (89). The core of this strategy lies in using macrophages as direct targets for “immune training,” establishing durable reparative functional programming at the transcriptomic level through local mRNA delivery—rather than a simple one-time polarization reversal. It must be cautiously assessed, however, that IL-4, as a cytokine broadly involved in *Th2* immune responses, has long-term effects on T-cell subsets and B-cell class switching upon local high-concentration delivery that await systematic evaluation.

As natural messengers for macrophage reprogramming, MSC-derived exosomes promote M1-to-M2 polarization by delivering molecules such as miR-21-5p to inhibit the *TLR4/NF-κB* pathway, whereas engineered M2-Exos enhance macrophage targeting through membrane modifications and load specific miRNAs to boost reprogramming efficiency (90). While their immunocompatibility as natural nanocarriers is a significant advantage, targeting specificity, cargo-loading efficiency, and scalable quality control remain technical hurdles for industrial translation.

### 5.5 Critical challenges in macrophage reprogramming: temporal control, specificity and translational gaps

The translational crux of macrophage reprogramming is precise temporal control: early M1 polarization is indispensable for pathogen clearance, and prematurely forced M2 polarization may paradoxically increase infection risk and promote fibrosis; the ideal intervention requires programmed phenotypic switching within the correct time window rather than simple polarization reversal (91). Currently, most reprogramming strategies still rely primarily on delivering exogenous factors and remain some distance from achieving true “*in situ* macrophage reprogramming”. Research on trained immunity in wound healing is still in its infancy, and the specific trained immunity pathways in different wound types await systematic elucidation. Moreover, while mannose- and integrin-targeted strategies exhibit good macrophage specificity *in vitro*, within the complex *in vivo* wound microenvironment, other cell types that highly express the same receptors (such as dendritic cells and endothelial cells) may compromise targeting efficiency and safety profiles, and systematic *in vivo* biodistribution and off-target evaluation data remain limited. In the future, integrating single-cell multi-omics technologies to resolve macrophage subset heterogeneity, developing temporally controllable nanodelivery systems with stringent macrophage specificity, and exploring combined metabolic and epigenetic intervention strategies will constitute the critical path for advancing macrophage reprogramming from proof-of-concept to clinical practice.

Macrophage reprogramming is the upstream key to breaking the vicious cycle of “inflammatory persistence–repair arrest”, which can fundamentally reverse the functional impairment of fibroblasts and keratinocytes in chronic wounds.

## 6 Challenges and prospects

The preceding sections have systematically established the four-dimensional technology toolbox for wound cell reprogramming and dissected the molecular mechanisms and intervention strategies for remodeling the identity of the three core repair cell types in chronic wounds—fibroblasts, keratinocytes, and macrophages (**Table 3**). These advances collectively lay a theoretical and preclinical foundation for shifting chronic wound treatment from passive supportive care to active, targeted reprogramming. However, clinical translation still faces three core bottlenecks that remain unresolved, and overcoming them will require both technological innovation and deeper mechanistic exploration.

**Table 3:** Summary of core molecular targets, reprogramming strategies, and translational status across the three wound cell types.

Cell type	Key dysfunction mechanisms	Reprogramming strategies	Representative molecules/tools	Translational stage	Key bottlenecks
Fibroblast	Piezo1/YAP/En1 mechano-epigenetic lock-in	Direct reprogramming; Small-molecule silencing	BMI1+FGFR2b (B2); siEn1@FibroMC	In vivo (mouse)	Temporal window; Heterogeneity
Keratinocyte	HDAC5/SETDB2/JMJD3 dysregulation; m^6^A defect; H3K18la	Endogenous activation; Exogenous generation	PITX1; B2 combination	In vivo (mouse)	Functional integrity; Long-term safety
Macrophage	TCA break; Succinate/Itaconate accumulation; KDM5A loss	Metabolic/epigenetic reprogramming; Trained immunity	Mannose-targeted microneedles; IL-4 mRNA LNPs; TPP-liposomes	In vivo (mouse/rat)	Temporal control; Off-target

### 6.1 Core challenges for clinical translation

#### 6.1.1 Delivery bottlenecks and insufficient platform integration

Delivery systems are the core prerequisite for the clinical translation of all reprogramming strategies, but their development is severely mismatched with the needs of wound reprogramming. Although *LNPs* and *AAV* vectors have been clinically validated in other disease areas, the complex microenvironment of open chronic wounds—including high levels of proteases, persistent bacterial biofilms, and drastic fluctuations in redox and pH—significantly reduces the stability, transfection efficiency, and targeting specificity of these delivery systems in situ (92, 93). More critically, existing delivery platforms are mostly single-function and single-drug designs, lacking the ability to sequentially respond to the dynamic healing process (inflammation → proliferation → remodeling), and cannot achieve the synergistic delivery of multiple reprogramming factors in a spatiotemporally controlled manner, which is the most direct technical barrier to the clinical transformation of multi-target combined reprogramming strategies.

#### 6.1.2 The core conflict between heterogeneity and precision

Single-cell and spatial multi-omics studies have confirmed the high functional heterogeneity of fibroblast and macrophage subpopulations in chronic wounds, and different subpopulations have completely opposite responses to the same reprogramming stimulus (94). However, almost all existing reprogramming strategies adopt a non-selective “blanket intervention” model, which not only leads to low reprogramming efficiency, but also brings potential off-target effects. Meanwhile, the activation of specific cell subpopulations is time-dependent during wound healing: early M1 macrophage polarization and myofibroblast activation are essential for bacterial clearance and wound contraction, and premature non-selective inhibition will directly lead to repair failure. At present, there is still a lack of reprogramming tools that can achieve single-cell-resolution targeting and dynamic temporal regulation, which is the core biological bottleneck restricting the precision of reprogramming intervention (95).

#### 6.1.3 Gaps in safety, models, and stratification

Safety is the primary threshold for clinical translation, but there is still a lack of long-term systematic evaluation data on the genomic integration risk of viral vector-mediated reprogramming, the epigenetic off-target effect of chemical reprogramming, and the long-term fate stability of reprogrammed cells *in vivo*. In addition, existing preclinical studies are highly dependent on mouse models, but mouse wound healing is dominated by wound contraction, which is fundamentally different from the re-epithelialization-dominated healing mode in humans; while porcine models are closer to human skin, their chronic wound model preparation has problems of long cycle, high cost, and poor reproducibility. More critically, the pathological microenvironments of diabetic foot ulcers, pressure ulcers, and venous stasis ulcers are significantly different, but existing reprogramming strategies rarely develop disease-specific intervention regimens based on the pathological characteristics of different wound types, and lack a standardized patient stratification system for precision reprogramming.

#### 6.1.4 Biological boundaries: intrinsic compensatory mechanisms and phenotypic reversion

A critical yet often underappreciated biological boundary of cell reprogramming in wounds is the inherent instability of the newly acquired cell identity once the initial therapeutic stimulus fades. The hostile, chronically inflamed wound microenvironment is not a passive recipient of reprogrammed cells but actively exerts counter-repressive pressure to restore the pathological status quo. One such mechanism is epigenetic memory and transcriptional drift. Studies in induced pluripotency have demonstrated that partially reprogrammed fibroblasts often harbor residual DNA methylation signatures at lineage-specific loci, which causes a strong propensity for the cell to revert to its original somatic identity upon cessation of transgene expression (96). Applied to wound repair, a fibroblast that has been reprogrammed to silence pro-fibrotic genes, such as Engrailed-1, could thus be driven back toward a scar-forming phenotype when the wound’s abundant TGF-β1 re-establishes suppressive chromatin marks at these loci (97). Equally important is niche-driven re-education. According to the macrophage niche model, tissue-resident macrophage identity is not fixed but continuously instructed by local growth factors and metabolites (98). In a chronic wound, even if pro-inflammatory macrophages are successfully trans-differentiated to a pro-reparative state, sustained stimulation by damage-associated molecular patterns such as HMGB1 and S100A8/A9, or by advanced glycation end-products in a diabetic milieu, can rapidly re-polarize them back to a hyper-inflammatory phenotype—a form of pathological trained immunity that paradoxically locks the cell in a dysregulated state (79). Finally, metabolic rebound poses a significant threat. Successful reprogramming of macrophages or fibroblasts toward a regenerative identity often depends on a shift from glycolysis to oxidative phosphorylation (99). However, the persistent hypoxia (HIF-1α stabilization) of a non-healing wound inevitably forces the reprogrammed cell back into a high-flux glycolytic state, effectively nullifying the therapeutic metabolic rewiring (100). Collectively, these compensatory mechanisms underscore that, without concurrently correcting the overarching pathological niche—through debridement, infection control, and normalization of the metabolic and hypoxic milieu—cell-intrinsic identity reconfiguration is intrinsically transient and prone to reversion.

#### 6.1.5 The halo effect: the unaddressed stromal ecosystem and crosstalk interference

ECM remodeling and wound resolution are emergent properties of a heterocellular stromal ecosystem extending far beyond the triad of fibroblasts, keratinocytes, and macrophages. An exclusive focus on these target cells risks a “halo effect,” where unmanipulated, niche-resident stromal populations compensate for or functionally bypass the intended reprogramming outcome. MSCs, both resident and circulating, act as paracrine gatekeepers of tissue homeostasis. They can sense and clear apoptotic bodies shed by successfully reprogrammed keratinocytes; this efferocytic process licenses MSCs to secrete anti-fibrotic and pro-regenerative factors such as TSG-6, thus amplifying the therapeutic benefit (101). Conversely, if the reprogramming strategy inadvertently induces cellular stress or senescence in the target fibroblast population, the resulting senescence-associated secretory phenotype can propagate maladaptive signals to neighboring healthy MSCs, converting them into fibrogenic effector cells and paradoxically accelerating pathological scarring (102). An even more direct source of fibrotic compensation is perivascular mural cells. Lineage-tracing studies have identified ADAM12+ pericytes as a major, autonomous source of myofibroblasts that drives fibrotic scar formation independently of dermal fibroblast activation (103). Consequently, a fibroblast-reprogramming therapy that yields near-perfect suppression of dermal myofibroblast conversion can still be completely subverted by the unimpeded differentiation of pericytes, leaving a major profibrotic engine operational. Furthermore, specialized stromal subclusters contribute to this compartmentalized biology. In hair-follicle-bearing wounds, for example, dermal adipocyte progenitors can transdifferentiate directly into myofibroblasts under aberrant Wnt/β-catenin signaling, representing a regeneration-competent cell type flipping into a scar-forming entity when the local niche is disrupted (104). Ignoring such adipocyte-to-myofibroblast transitions means that a reprogramming cocktail targeting canonical fibroblasts would be biologically irrelevant in anatomical zones where adipocytes are the primary fibrotic effectors. Similarly, dysfunctional lymphatic endothelial cells can undermine macrophage reprogramming by failing to drain interstitial inflammatory infiltrate, thus sustaining the chronic inflammatory signaling that inevitably pushes macrophages back toward a pathologic activation state (105). Therefore, a truly durable cell-identity reprogramming strategy for chronic wounds necessitates an ecosystem-level design, where the reprogrammed state of the target cell is stabilized by simultaneous modulation of the MSC niche, pericyte recruitment, and lymphatic clearance pathways.

#### 6.1.6 Bridging the mouse-to-human translational gap with human-derived platforms

The vast majority of mechanistic and therapeutic evidence cited in this review derives from murine models, yet rodent wound healing is predominantly driven by panniculus carnosus-mediated contraction, whereas human repair relies on re-epithelialization and granulation tissue formation (106). This fundamental divergence means that reprogramming efficiencies, lineage conversion stability, and functional rescue observed in mice may not faithfully translate to clinical settings. To bridge this gap, we must critically recalibrate our translational expectations against data obtained from human-derived platforms. Human skin organoids, generated entirely from human pluripotent stem cells, recapitulate the full thickness of native skin, including stratified epidermis, dermis, and appendages such as hair follicles (107). These organoids provide a genetically tractable, humanized system in which the identity reprogramming of fibroblasts, keratinocytes, and macrophages can be traced in a tissue context that mirrors human architecture and maturation. Similarly, three-dimensional organotypic skin rafts incorporating primary human keratinocytes, fibroblasts, and immune cells have been engineered to mimic the chronic wound microenvironment, including hyperglycemia, oxidative stress, and persistent inflammation. When challenged with diabetic-like conditions, these constructs allow direct assessment of whether a reprogrammed cell can maintain its new identity and exert pro-healing functions under sustained pathological stress, thereby revealing potential phenotypic reversion or compensatory resistance that mouse models cannot easily capture. Microphysiological skin-on-a-chip platforms further complement these models by integrating dynamic media flow, mechanical stretching, and real-time live-cell imaging, enabling quantitative evaluation of stem cell mobilization, re-epithelialization kinetics, and the spatiotemporal effects of intelligent delivery systems (108). By iteratively cross-validating reprogramming strategies across mouse models and these human-derived systems, the field can more robustly triage which interventions are ready for clinical translation and which require further optimization. The systematic incorporation of patient-specific organotypic models and chip-based platforms into preclinical pipelines is not merely an optional refinement but an essential step toward the “personalized diagnosis → intelligent sequential delivery → closed-loop healing monitoring” paradigm envisioned in this review.

#### 6.1.7 Immunological consequences of stromal identity reprogramming

Stromal cells are not merely passive scaffolds but active regulators of both innate and adaptive immunity within the wound microenvironment. Dermal fibroblasts, for instance, are prolific sources of chemokines such as CCL2 and CXCL1 that direct myeloid cell recruitment, and of cytokines including IL-6 and TGF-β1 that polarize infiltrating monocytes toward profibrotic macrophages and shape T-cell effector functions (109). Consequently, rewriting the molecular identity of scar-prone fibroblasts toward a regenerative phenotype fundamentally alters the local cytokine and chemokine landscape. Landmark studies have demonstrated that preventing Engrailed-1 activation in wound fibroblasts not only blocks pathological extracellular matrix deposition but also reconfigures the immune infiltrate—reducing monocyte-derived macrophage accumulation and shifting the balance from a pro-inflammatory to a pro-resolving milieu (110). By extension, reprogramming pericytes or adipocyte progenitors away from a myofibroblastic fate would be predicted to attenuate the release of fibrogenic and immunostimulatory factors that sustain Th2-skewed responses and impair regulatory T-cell function. Through the restoration of a non-fibrotic stromal identity, these interventions may indirectly promote immune tolerance by enhancing Treg recruitment and fostering local production of immunosuppressive mediators such as prostaglandin E_2_ and indoleamine 2, 3-dioxygenase (111). Thus, cell identity reprogramming in the wound stroma serves as a dual therapeutic lever: it corrects the structural pathology while simultaneously breaking the feed-forward loop of stromal-immune crosstalk that perpetuates chronic inflammation.

### 6.2 Future directions and breakthrough paths

#### 6.2.1 Single-cell multi-omics navigation and AI-driven precision reprogramming design

To address the core conflict between cellular heterogeneity and insufficient intervention precision, future research should integrate single-cell transcriptomics, epigenomics, spatial omics, and metabolomics to construct a high-resolution spatiotemporal dynamic map and cell-cell communication network of chronic wounds. This map will accurately locate the pathogenic cell subpopulations that drive poor healing, and identify the core regulatory nodes of cell fate decision-making. On this basis, deep learning models can be trained on multi-omics big data to predict the optimal combination of reprogramming factors, small molecule formulations, and delivery timing for specific pathogenic cell subpopulations, so as to develop individualized precision reprogramming regimens for different wound types and patient populations.

#### 6.2.2 Small molecule-based reprogramming system and closed-loop intelligent delivery materials

To improve the safety and clinical accessibility of reprogramming strategies, priority should be given to the development of drug repurposing and chemical reprogramming technologies, and a systematic small molecule library for wound repair reprogramming should be established by screening FDA-approved drugs and novel small molecule compounds with clear epigenetic/metabolic regulatory targets. For the delivery bottleneck, a new generation of closed-loop intelligent biomaterials integrating “real-time sensing - intelligent decision-making - on-demand delivery” should be developed. Such materials can monitor the pH, *ROS*, and enzyme activity of the wound microenvironment in real time, and release anti-inflammatory factors, pro-angiogenic factors, or reprogramming regulators in a stage-specific and dose-controlled manner according to the dynamic healing process, so as to achieve full-course sequential precise intervention of wound healing.

#### 6.2.3 Trained immunity-based immune resetting and *in situ* multi-lineage reprogramming

To fundamentally solve the core problems of persistent chronic inflammation and insufficient repair cell sources, two frontier directions should be focused on. First, harnessing the concept of trained immunity to develop a “benign retraining” strategy targeting bone marrow progenitor cells, which can erase the pro-inflammatory epigenetic memory of innate immune cells induced by hyperglycemia and other pathological factors, and reset the immune function of chronic wounds from the hematopoietic source. Second, expanding the boundary of *in vivo* direct lineage reprogramming, to explore the feasibility of using composite sequential signals to reprogram wound-resident fibroblasts into complete skin organoids with epidermal, dermal, vascular, and even hair follicle structures, so as to achieve full-thickness skin regeneration *in situ* for large-area wounds.

## 7 Conclusion

In summary, effective restoration of chronic wound healing requires the coordinated reprogramming of the three principal resident cell populations—fibroblasts, keratinocytes, and macrophages—and their dynamic intercellular crosstalk. Fibroblasts, traditionally viewed as scar-forming myofibroblasts, can be steered toward a regenerative phenotype by attenuating *TGF-β* signaling and reversing excessive extracellular matrix deposition, thereby restoring dermal architecture (34). Keratinocytes, as the primary drivers of re-epithelialization, rely on a finely tuned network of epitranscriptomic modifications—including m6A methylation and lactylation-driven epigenetic switches—that balance proliferation, migration, and differentiation, offering actionable targets for accelerating epidermal barrier reconstruction (63, 64). Macrophages, the central immune sentinels, must transition from a destructive M1 state to a reparative M2 phenotype, a shift that is currently hindered by a self-reinforcing metabolic–epigenetic loop driven by succinate-mediated *HIF-1α* stabilization and *KDM5A*-dependent chromatin silencing at M2 gene loci (72, 76). Notably, the recent discovery that non-immune resident cells (pericytes) can impose a durable “trained immunity” on macrophages via *SASP* factors and mitochondrial transfer reveals that macrophage polarization is not merely cell-autonomous but is extrinsically enforced by the stromal compartment (78–80).

Crucially, these cell types do not operate in isolation. Fibroblasts provide the provisional matrix for keratinocyte migration and secrete paracrine factors that influence macrophage polarity; keratinocytes release alarmins that shape the immune milieu; and macrophages govern both fibroblast activation and keratinocyte proliferation through a rich secretory repertoire. Such interdependent signaling demands a multi-cellular reprogramming strategy that delivers the right molecular cue to the right cell at the right time. This is now becoming feasible through intelligent delivery platforms—for instance, cascade-targeting pH/ROS-responsive microneedle patches that allow sequential release of metabolic reprogrammers to macrophages followed by pro-regenerative signals to fibroblasts and keratinocytes (84), and triple-targeting miRNA-loaded core–shell nanoparticle–hydrogel composites that simultaneously modulate multiple cell types within the wound (32).

Looking forward, three emerging frontiers will propel the field from proof-of-concept toward a precision systems-engineering paradigm of “personalized diagnosis → intelligent sequential delivery → closed-loop healing monitoring.” First, single-cell multi-omics and spatially resolved transcriptomics will deconvolve the cell–cell communication networks at unprecedented resolution, enabling the discovery of combinatorial targets for precision reprogramming (67). Second, AI-driven temporally programmed smart materials will integrate real-time wound signals (pH, ROS, protease activity) to autonomously adjust the release kinetics of multiple therapeutic agents, thereby achieving true chrono-controlled delivery. Third, trained immunity offers a novel avenue to durably reset the innate immune memory of macrophages and their stromal instructors, potentially preventing chronicity through a single intervention (78, 80). Ultimately, translating these innovations into clinical reality will require rigorous validation in clinically relevant large-animal models and patient-stratified trials that address the core bottlenecks of safety, cellular heterogeneity, and spatiotemporal precision discussed throughout this review. Such a holistic, multi-cell, multi-technology approach holds the promise of transforming the management of chronic non-healing wounds from palliative care into curative regeneration.
